# The Chemistry of Behind the UV-Curable Nail Polishes

**DOI:** 10.3390/polym17091166

**Published:** 2025-04-25

**Authors:** Inese Mieriņa, Zane Grigale-Sorocina, Ingmars Birks

**Affiliations:** 1 Institute of Chemistry and Chemical Technology, Faculty of Natural Sciences and Technology, Riga Technical University, P.Valdena Str. 3, LV-1048 Riga, Latvia; 2 R&D, Kinetics Nail Systems, Kurzemes Prospekts 3K, LV-1067 Riga, Latvia; zane.grigale@kineticsbeauty.com (Z.G.-S.); ingmars.birks@kineticsbeauty.com (I.B.)

**Keywords:** nail polish, UV curable, acrylates, bio-based compositions

## Abstract

As far as history tells, people have set efforts both to improve the conditions and to change the visual outfit of the skin, nails, and hair. The first information on nail cosmetics is found in ancient China and Egypt, where various nature-derived compositions were used for changing the colour of the nails. Nowadays more mechanically and chemically durable systems for nail polishes are elaborated. This review focuses on the latest achievements in the field of UV-curable nail polishes. Herein, the polymerization mechanisms of various systems (acrylates, as well as epoxides and thiols) occurring in nail polishes are described. Besides plausible side reactions of the polymerization process are characterized. Thus, the main drawbacks for forming a uniform, perfect layer are illuminated. For effective curing, the choice of photoinitiators may be crucial; thus, various types of photoinitiators as well as their main advantages and disadvantages are characterized. Ensuring effective adhesion between the substrate (human nail) and the polymer film is one of the challenges for the nail polish industry—thus the plausible interactions between the adhesion promoters and the keratin are described. Regarding the film-forming agents, a comprehensive overview of the composition of the traditional UV-curing nail polishes is provided, but the main emphasis is devoted to alternative, nature-derived film-forming agents that could introduce renewable resources into nail cosmetics. Additionally, this review gives short insight into the latest innovations in UV-curing nail cosmetics, like (1) nail polishes with improved pealability, (2) covalently polymer-bonded dyes and photoinitiators, thus reducing the release of the low-molecular compounds or their degradation products, and (3) UV-curing nail polishes as delivery systems for nail treatment medicine.

## 1. Introduction

The global market of beauty and personal care products is constantly growing [1]: the market for skincare, fragrance, makeup, and haircare products was around 430 billion dollars in 2022; it is expected that more than 550 billion will be reached in a five-year period, respectively in 2027 [2]. The total market for nail gels was about USD 55.6 million dollars in 2023. The forecast for the year 2030 foresees the growth of the market up to USD 88 million dollars [3].

Nail polish, or nail lacquer, has a long-standing history, with the first attempts already in ancient China and Egypt when nature-based components were applied on the nails. The development of nail polishes in the form of today was started in the beginning of the 20th century when various film-forming polymers were available. These nail polishes should be considered as a solution of polymers that, upon evaporation of the solvent, forms a thin layer. The third significant turning point in the development of nail polishes is inspired by dentist Fred Slack [4], who used dental acrylate to fix his nail.

The modern composition of nail polishes (also referred to as nail glues or nail lacquers) is a complex formulation consisting of various film-forming agents as well as additives (like leveling agents, wetting agents, antifoaming agents, colors, minerals, etc.) that provide the desired mechanical, physical, and sensory properties for the product. The extensive growth of the market has triggered new studies on both health issues and the improvement of the composition of the product. Herein, we have turned our focus towards UV-curable nail coatings—from the chemistry viewpoint, these products can be classified as “acrylate chemistry”.

The main drawbacks highlighted for acrylates from the health perspective are attributed to plausible contact dermatitis and occupational diseases: some case studies have revealed allergic contact dermatitis for isobornyl acrylate, 2-hydroxyethyl methacrylate, and ethyl acrylate [5]. It is reported that nail dystrophy [6] or pseudo-psoriathic nails [7] may be developed under exposure to acrylate nail products. Additionally, acrylate monomers can cause asthma by respiratory sensitization [8]. These aspects are analyzed in several review articles [representative examples: [5,9,10,11,12,13]. Another plausible issue of health risks is associated with exposure of the skin and nails to UV or LED irradiations. These risks should be taken into account; however, it is nearly impossible to compare them for various cases, such as the light source and the maximum wavelength, as well as exposure time, which strongly depends both on the equipment and the nail products applied. It is speculated that some UV-induced DNA damage could occur. It should be admitted that the current status quo remains that when UV curing is done properly, the potential risk is acceptably low [14]. Contrary to when one is subjected to improper over-exposure (under unknown quality equipment), phototoxic effects may occur [15]. Some studies demonstrate that sunscreens could be useful for improving the keratinocyte viability [16]. A part of the health issues is more plausible for the client, a part—for the manicurist, and some—for both.

This review covers mainly the chemistry behind UV-curable nail coatings, highlighting the latest improvements and tendencies. Several UV-curable nail polishes with the desired properties are available on the market. However, studies on new innovations and improvements for these products are still up to date. These trends are mainly covered by patent materials—more than a hundred patents covering various UV-curing nail cosmetic products are available in the Espacenet database. More than 60% of all the products are classical compositions containing polymerizable acrylate monomers and oligomers, as well as various polymers. These formulations will not be discussed in detail herein. Some of the innovations also involve modifications of these compositions to improve the properties for the nail polish films, which are discussed within this review.

Further significant breakthroughs for these products leading to remarkably improved properties are really challenging and related to relatively small changes in the formulations leading to dramatic evolution of the product. To achieve this challenge, understanding the chemistry occurring behind the curing process and the plausible interactions among various ingredients is essential. So, this review in-depth focuses on the reaction mechanism occurring during the UV-curing process and plausible side reactions. Comprehension of these findings can be essential for designing more efficient formulations with improved properties and resistance against various side processes. We have focused on new and prospective UV-curable compounds and products that might be an alternative to the classical UV-curing nail products. The main emphasis turns to the film-forming agents; however, some aspects of the additives for modifying the adhesion between the surface of the nail and the coating are analyzed, too.

## 2. An Overview of the Polymerization Reactions

Regarding the polymerization mechanism, the cationic and radical polymerization processes are applied. UV-curing nail compositions consist of polymerizable monomers or oligomers which are later exposed to light, and polymerization occurs. The polymerization efficiency strongly depends on the composition of the nail polish gel as well as photoinitiators. A brief overview of various systems is summarized in Table 1.

### 2.1. Acrylate Chemistry

**Figure 1:**
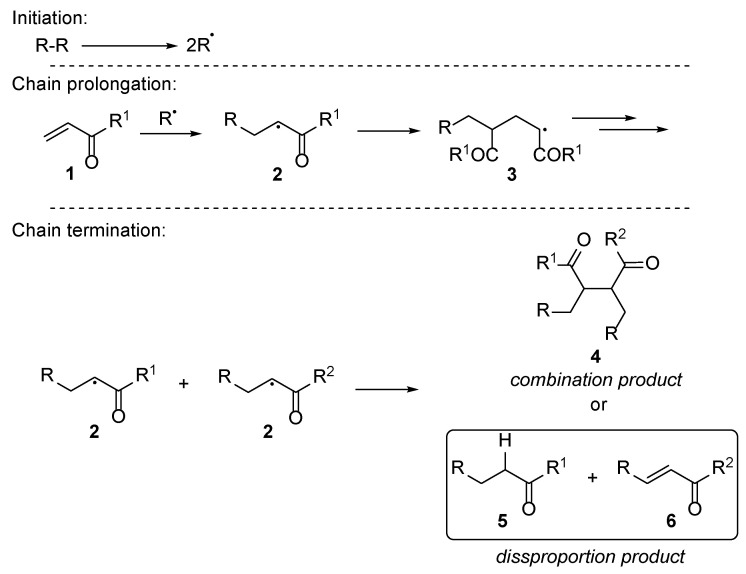
General scheme of the radical-mediated acrylate polymerization.

**Figure 2:**
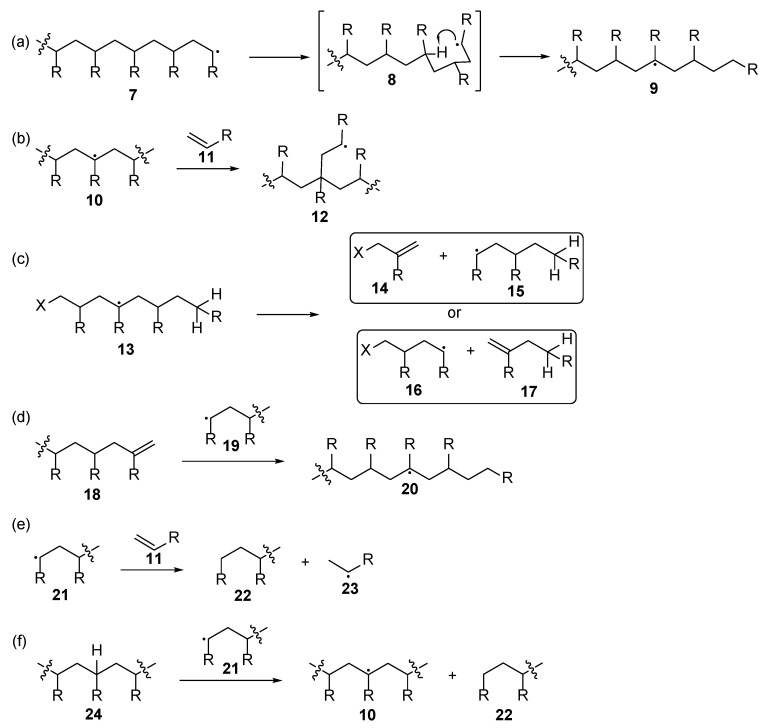
Various plausible side reactions of the acrylate polymerization: (**a**) 1,5-migration (also called “backbiting”); (**b**) mid-chain radical propagation; (**c**) β-scission; (**d**) macromonomer propagation; (**e**) chain transfer to the monomer; (**f**) chain transfer to polymer.

Chemistry and mechanistic insight behind the acrylate polymers are reviewed in detail by Ballard and Asua [17]. Briefly, the process involves initiation of free radicals, chain prolongation and termination of the polymerization chain (Figure 1). The polymerization reaction may be interrupted both by combination and disproportion leading to the corresponding products **4** and **5**, **6**. Studies on model substrates have revealed that when the reaction occurs under UV irradiation at relatively low temperatures, disproportion products **5** and **6** exclusively are formed. The increase in the temperature promotes formation of combination product **4** (around 4% are formed at 60 °C) and various side reactions (Figure 2) [18]. The application of the nail polishes foreseen that the polymerization process should occur with relatively low heat increase in order not to cause discomfort during the application process. Thus, it could be expected that the disproportion process is dominant herein.

The idealized mechanism foreseen long polyacrylate chain formation with even distribution of the molecular size for the formed polymer. However, it is observed that a queue of various defects (Figure 2) may occur, thus leading to polymers with lower molecular weight, additional cross-linkage, etc. Firstly, 1,5-migration of the hydrogen atom from the terminal position in compound **7** may occur, thus leading to mid-chain radical **9**. The last one can undergo additional chain propagation through the reaction of additional monomer **11**, thus leading to branched polymers **12**. Moreover, *β*-scission may undergo, and short chain polymers **14**–**17** are formed as a result. These newly derived monomers may be subjected to new polymer chain-growth reactions leading to branched polymeric structures [19]. From one side, it could be expected that polymers with increased cross-linking could demonstrate improved properties for nail polishes due to, e.g., reduced solubility. On the other side, these secondary reactions lead to an increase in the polymers with reduced molecular weight that could result with reduced mechanical properties of the nail coating.

UV-curing processes are mediated by photoinitiators—light-sensitive compounds that, upon irradiation, tend to form free radicals. The latter ones initiate the polymerization reaction chain. The limiting factor for them is perfect coincidence between the absorption bands of the photoinitiator and the emission spectra of the light source. These compounds may act under both UV and visible light. However, the application of the products limits the usage of visible light colorless compounds; otherwise, the properties of the final product may be influenced, firstly due to the unpredictable color changes and secondly due to the insufficient absorption of the light caused by the competitive light interaction with the dyes introduced in the formulations of the product. Thus, the most suitable photoinitiators are colorless compounds that are excited by UV irradiation. On the other hand, it should be taken into consideration that the shorter the irradiation wavelength, the greater the effect of the light diffusion, and thus the penetration depth is reduced [20]. Although the layers of the nail polish coating are rather thin, various particles responsible for the color and effects of the outfit for the coating may remarkably encumber excellent curing of the layer.

**Figure 3:**
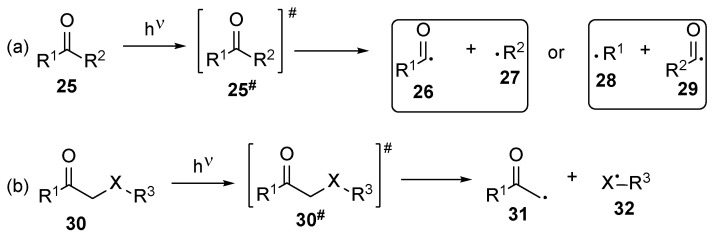
Generation of free radicals from type I photoinitiators: (**a**) α-scission and (**b**) β-scission.

Two types of photoinitiators are known—type I and type II [21] (known also as the Norrish type I and II photoinitiators [22]). Both photoinitiators differ from one another through the mechanism of generating the free radicals. The type I photoinitiators **25** and **30** (Figure 3), in the presence of suitable irradiation, undergo homolytic C-C bond scission between the carbonyl group and α-carbon, also known as alpha or Norrish scission (Figure 3a) [5,15], leading to acyl **26** and **29** and alkyl radicals **27** and **28**. Alternatively, *β*-scission (Figure 3b), when the bond between the α-carbonyl next to the carbonyl group and the heteroatom is shrunk, may occur [23]. The energy required for free radical generation from type I photoinitiators is rather high, and high-energy light sources are necessary [24].

**Figure 4:**
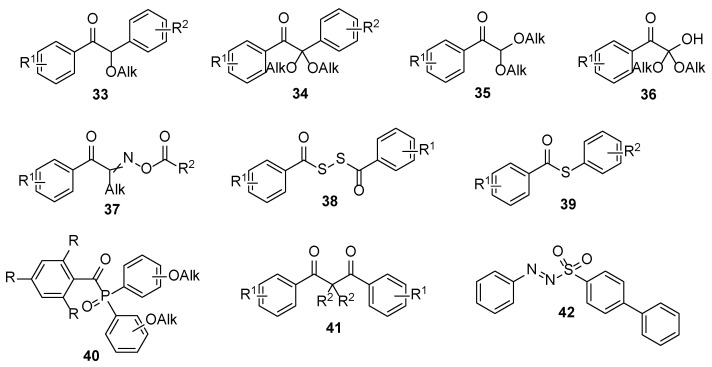
General structures of type I photoinitiators.

Well-known type I photoinitiators are various *α*-(di)alkoxydeoxybenzoins **33** and **34**, *α*,*α*-alkoxyacetophenones **35** and **36**, oximinoketones **37**, dibenzoyl disulphides **38**, thiobenzoates **39**, acyl phosphine oxides **40**, dibenzoylmetanes **41**, and azo-compounds **42** (Figure 4) [25]. It should be noted that the acyl phosphine oxides are the most widely used for nail products.

**Figure 5:**
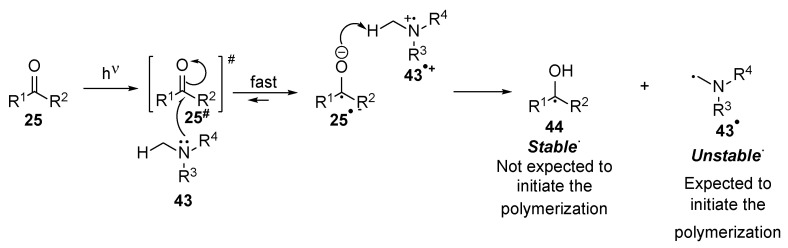
Generation of free radicals from type II photoinitiators.

Contrary to type II photoinitiators **25** are excited and then through several transformations, the radicals **43^·^** and **44** are generated (Figure 5). Firstly, the interaction between the species **25 ^#^** and an amine **43** or alcohol led to radical anion **25^·−^**. Finally, the proton abstraction from the radical cation **43^·+^** leads to ketyl radical **44** and amine radical **43^·^** [21]. It should be noted that the ketyl radical **44** due to resonance, is rather stable and sterically hindered; thus, it is not expected that the ketyl radical **44** induces the polymerization reaction. The radical **43^·^** formed from the co-initiator is responsible for the initiation of the polymerization reaction [26].

**Figure 6:**
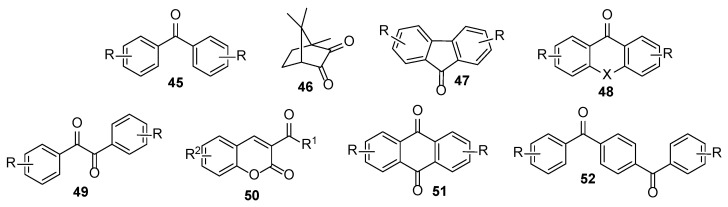
General structures of type II photoinitiators.

Among type II photoinitiators, various ketones like benzophenones **45** and **52**, camphor derivatives **46**, fluorenones **47**, xanthones **48**, benzils **49**, acyl coumarines **50**, and anthraquinones **51** are well established (Figure 6) [25].

**Figure 7:**
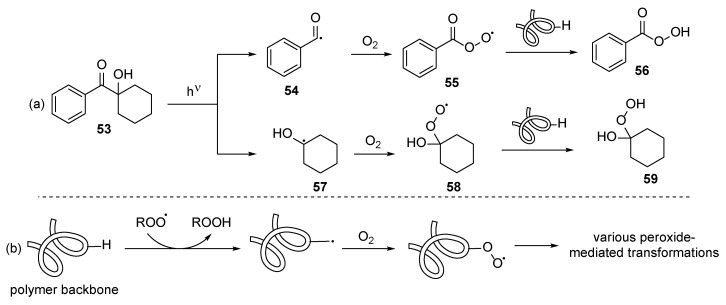
Plausible oxidation-caused side reaction: (**a**) oxidation of the photoinitiator species and (**b**) oxidation of the polymer backbone.

The main challenge for radical polymerization is the inhibition of radical reactions by oxygen. The traditionally used photoinitiators **53** tend to form peroxides **56** and **59** when they react with oxygen. As a result, the newly formed peroxyl radicals **55** and **58**, instead of undergoing acrylate polymerization, tend to abstract a hydrogen from the polymer backbone (Figure 7a). Later, the polymer backbone undergoes further oxidation reactions leading to peroxide-decorated polymers (Figure 7b) [27]. Additionally, due to the high reactivity of the hydroperoxides, both various additional cross-linking and cleavage of the polymer chain may occur.

For industrial acrylate coating processes, the impact of the oxygen and incomplete polymerization can be diminished by an air-free atmosphere and a rather high intensity of the UV light. It is hard to imagine an oxygen-free environment for applying nail polishes; thus, oxygen-induced inhibition of polymerization is reasonable. As a result, the main drawbacks herein are (1) a sticky layer of small-molecular products on the surface, which should be later removed, and, more serious, (2) these small molecular compounds can migrate from the film in the human body. A straightforward solution is an increase in the light intensity and the concentration of the photoinitiator; thus, the amount of unpolymerized residues may be reduced by up to 6-fold [28]. The sensory tests demonstrate that a reasonable increase in the amount of photoinitiator and light intensity is not acceptable for rapid polymerization on the nail surface. Thus, studies of effective photoinitiators and/or their mixtures turn out to be important players for effective UV-curing processes.

To solve the oxygen-inhibited photo-curing, synergistic photoinitiator compositions containing type I and type II photoinitiators are used. Usually, these compositions are accompanied by copolymerizable amines, which later are incorporated in the structure of the polymer [29].

### 2.2. Epoxide Chemistry

**Figure 8:**
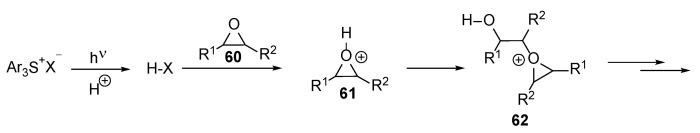
General scheme of epoxide polymerization.

**Figure 9:**
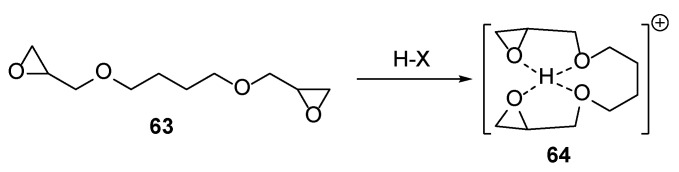
Formation of the secondary oxonium intermediates.

Undesired oxygen-caused side reactions may be overcome via the cationic polymerization process. Typically, cationic polymerization is applied for epoxide hardening [30,31]; however, examples where other oxygen heterocycles like oxetane or double bond systems like vinyl ether derivatives are cured under this mechanism are known, too [31]. Some of the described nail polish compositions may also contain epoxide moieties. The cationic ring-opening process is postulated for the epoxide polymerization [30]. The reaction sequence involves generation of UV-light-mediated Bronsted acid from the photoinitiators, called photoacids, followed by protonation of the epoxide ring and oxirane cleavage (Figure 8). It is speculated that cationic polymerization may involve secondary oxonium intermediates **64** (Figure 9). These species are rather stable at low temperatures, but later they may turn into active centers, and curing is self-catalyzed [32]. When the reaction is run without an additional nucleophile, an active chain end mechanism is postulated. On the contrary, when the polymerization proceeds in the mixture containing additional nucleophiles like alcohols, a competitive activated monomer mechanism may occur [33]. The cationic mechanism is supported for double bond systems bearing electron-donating substituents due to increased stability of such cationic intermediates, too [34].

The main disadvantage of such systems is that the method can be applied in a thin layer, not in a bulk reactor [31]. Although thin layers are typical objects for applying nail polishes, a part of such cationically polymerizable systems is developed in such a manner that the heat developed during the curing is high enough to develop a heat-induced polymerization process [31]. Thus, when such compositions are elaborated for nail polish applications, the plausible thermal reaction should be taken into consideration.

**Figure 10:**
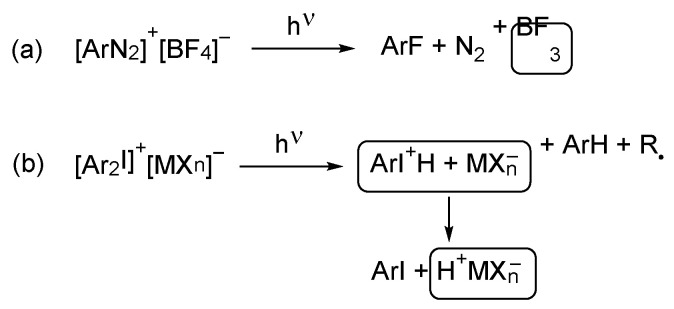
Generation of (**a**) the Lewis acid or (**b**) the Bronsted acid from ionic photoacids.

All photoacid generators are classified as ionic and non-ionic species. The first group contains a cationic motif and a metal halide species acting as an anionic counter anion (Figure 10). These compounds usually dissociate via a radical pathway, and a proton from surrounding ingredients may be extracted; thus, either a Bronsted or a Lewis acid is generated. Typical examples of such photoacids are diazonium, halonium, sulfonium, or phosphonium compounds [35]: the cationic part is responsible for the photochemical properties (like absorption, wavelength, etc.); the anionic part is responsible for the overall acidity of the formed acid [36].

**Figure 11:**
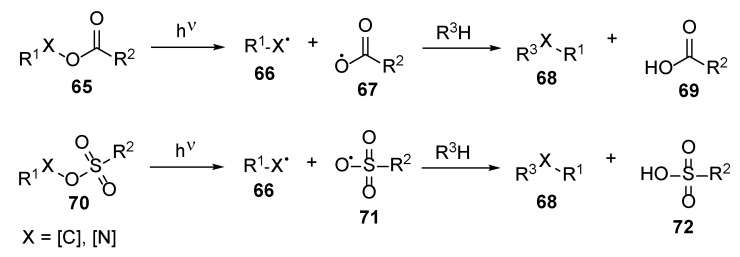
Generation of the Bronsted acids from the non-ionic photoacids.

Non-ionic species should be considered a newer group of photoacid precursors. The acidic species are generated through photolytic homolytic dissociation of C-O, S-O, or N-O bonds. Often these reactions are accompanied by extraction of the hydrogen from some protic ingredient of the composition (Figure 11). Structures acting as non-ionic photoacid generators are benzyl carboxylates and sulfonates, as well as imino esters [35]. Widely used photoacids are both (1) diaryldiazonium salts, which upon irradiation elaborate nitrogen and the Lewis acid, and (2) triarylsulphonium and diphenyliodonium salts, which act as the Bronsted acid precursors [37]. Another group of photoacids are spiropyrans, which upon irradiation may undergo reversible ring cleavage—closure elaborating free acid [35].

**Figure 12:**
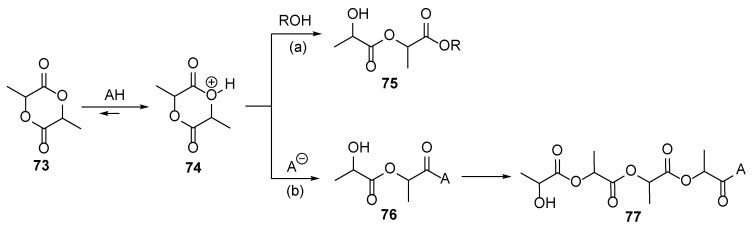
General scheme of the lactide polymerization: (**a**) in the presence of external nucleophile and (**b**) without additional nucleophile.

Unfortunately, epoxide and oxetane derivative hardening on human nails could be rather harmful due to the high reactivity of these heterocycles: these heterocycles could undergo nucleophilic attack from various species, including various biomolecules in the human body like amino acid residues, DNA bases, etc., leading both to plausible mutagenic effects and covalent bonding of the layer with the nail surface. On the other hand, some other pre-polymers, like lactides, could be promising candidates for such reactions. Up to date, the polymerization of lactides **73** is well established in the presence of the classical Bronsted and Lewis acids (Figure 12) [38]. These studies give strong evidence that lactides may be cured under irradiation when suitable photoacid is used. It should be admitted that generally polymerization is faster when the cationic mechanism is involved. Additionally, in some cases, high temperatures are not required, too [34].

An advantage of the cationic mechanism over the radical mechanism, besides the resistance towards oxygen-caused side reactions, is lower shrinkage of the material. Thus, it may be expected that the material would demonstrate better adhesion to various surfaces [39].

Nowadays efforts are also turned towards shifting from the UV-light-mediated processes to visible-light-mediated cationic polymerization. Herein a typical catalytic system consists of a photosensitizer, a carbocation source, and an oxidant: it is expected that the photosensitizer will be excited by the visible light; later the excited species forms a carbon radical, which upon oxidation leads to the carbocation [40]. Up to date, the application of visible light cationic polymerization is rather challenging for nail products, as one of the most important aspects of them is aesthetic issues, and the color agents may be incompatible with the catalytic systems. On the other hand, the research of various photosensitizers is topical, and photosensitizers suitable for these applications without the necessity of UV irradiation may be elaborated.

**Figure 13:**
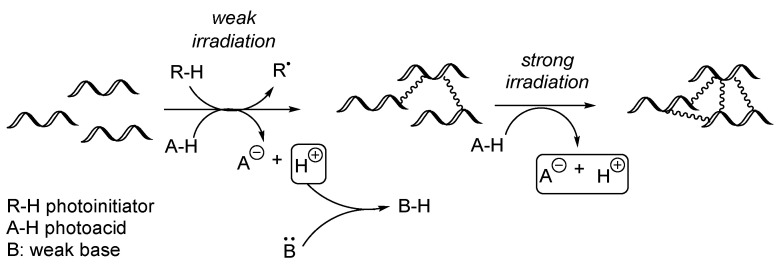
Sequential UV-curing through radical and cationic process.

To combine advantages for both cationic and radical polymerization, compositions containing both radical-curable ingredients (typically acrylates) and cationically curable moieties (e.g., epoxides) are elaborated, e.g., for photolithography purposes. The simplest systems are implemented in a “batch” mode when simultaneously the curing through both mechanisms is achieved [41]. Moreover, this procedure may be improved for switching from one mechanism to another. Such a composition combines in the same system type I or II photoinitiators, photoacids, and a weak base. It is postulated that a simple variation of the light dosage could allow switching the mechanism from the free radical polymerization to the cationic process (Figure 13). Thus, the properties of the product may be varied [42]. With a future process design perspective, this could also be a tool for improved nail polishes when, firstly, the radical process could occur, and later the curing could be even strengthened through the cationic polymerization. Thus, the surface of the nail polish could be even more durable, and the amount of the sticky unpolymerized oligomers could be reduced.

### 2.3. Thiol-Ene Chemistry

**Figure 14:**
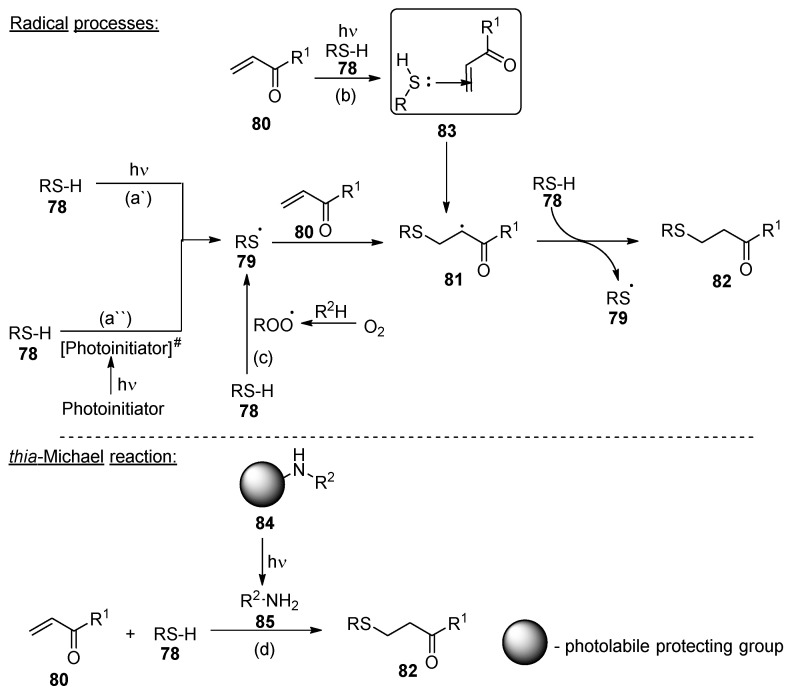
General scheme of the thiol-ene polymerization via (**a**) radical mediated step-growth mechanism; (**b**) charge transfer complex; (**c**) oxidation involved process; (**d**) thia-Michael reaction.

An alternative for reducing the oxygen-caused inhibition of light-cured acrylate polymerization uses thiol additives—some of the established UV-curable nail polish compositions also contain thiol derivatives as the film-forming agents [43,44]. Such photopolymerization undergoes a thiol-ene reaction. Although the reaction is not inhibited by oxygen, the presence of it slightly reduces the polymerization rate [45]. Thiol-ene systems typically consist of monomers containing several thiol **78** or double bond **80** functionalities. Electron-rich double bonds are more reactive towards the thiyl radicals **79**, and the reactivity order is assigned as follows: vinyl ethers > allyl ethers > acrylates [46]. The polymerization mechanism is not fully clear in detail. However, two main pathways are discussed (Figure 14) [45]. The thiol-ene radical polymerization process is reviewed by Lowe [47]: the process involves a generation of sulphur radical **79**, which later reacts with the carbon-carbon double bond **80** (Figure 14). Contrary to the chain growth mechanism assumed in acrylate systems, herein it is postulated that the polymerization proceeds via a radical-mediated step-growth mechanism (Figure 14a) leading to the corresponding thio-ethers **82** [48]. The radical-step growth mechanism ensures a highly homogenous network of material [48,49]. An alternative mechanism foresees intramolecular interaction between the thiol and the double bond, and the reaction might undergo via the charge transfer complex **83** (Figure 14b) [45]. The reduced oxygen impact on the thiol-ene polymerization is attributed to various stabilities of the involved radical species: (1) the reactivity of peroxy radicals is reduced in comparison to carbon radicals, thus the polymerization rate is remarkably diminished, and (2) contrary to when thiol-containing systems are polymerized, the thiol demonstrates a dual role: besides generating thiyl radicals **79**, it may act as an antioxidant through simultaneously scavenging peroxyl radicals, and the newly borne thiyl radical **79** may initiate new polymerization chains (Figure 14c) [45].

Contrary to the acrylate and epoxide polymerization, herein a nucleophilic addition known as the thia-Michael reaction may be under consideration (Figure 14d). For this mechanism, photobases **84** (or photonucleophiles) are required [50]: the first principles were demonstrated in 2013, when 2-nitrobenzyl functionality was linked to the amines, and later, upon irradiation, the amine base was released, and the thia-Michael reaction may occur [50]. However, some of the photobases may form carcinogenic nitroso species. Thus, application of the photobases and plausible side products should be considered before the elaboration of these compositions.

Contrary to acrylate or epoxide examples, thiol-ene radical light-mediated polymerization may occur both with (Figure 14a″) and without (Figure 14a′) the photoinitiator (a typical example is benzophenones). Photoinitiator-free processes are preferred over those involving photoinitiators mainly due to the reduced yellowness of the polymer caused by the residues of the photoinitiators [45]. The thiol-ene reaction may be initiated by both type I and type II photoinitiators. When type I photoinitiators are used, both thiol-ene reactions may occur and a typical acrylate polymerization may be accelerated. Contrary to when type II photoinitiators are applied, the thiyl radical **79** is generated along with the sterically hindered ketyl radicals. The last ones are reasonably less reactive towards the double bond system [51]; thus, it can be expected that type II photoinitiators are preferable when the radical-mediated step-growth mechanism should be ensured.

The main drawback for such systems is unpleasant odor derived from the thiols, as well as the low stability during storage [49]: the insufficient stability arises mainly from free radical transformations generated from the thiol-ene charge transfer complexes **83** [50]. The stability of the compositions may be improved by (1) modification of the formulations (it is well known that the secondary thiols are more stable than the primary) and (2) phenol-type antioxidant additives, which transfer the generated free radicals into more stable compounds [50].

On the other hand, nail polish compositions containing thiol derivatives are characterized by a shortened rate of polymerization [44,52]. Moreover, these films are characterized by high gloss, good adhesion [53], and reduced shrinkage [46]. Additionally, the nail keratin contains various elements, including sulphur-containing amino acids, and some competing side reactions between the nail polish and the nail surface may be expected.

## 3. Acrylate-Based Compositions as the Film-Forming Agents

### 3.1. Petroleum-Based Acrylate Compositions

A typical UV-curing nail polish gel is a composition of various petroleum-based acrylate/methacrylate derivatives. Acrylate-based UV-curable nail coatings are characterized by long wearing time and gloss. The latest achievements are devoted both to client-safer compositions, thus reducing irritation risk caused by acrylates or solvents due to soaking during the removal of nail coatings, as well as bio-based compositions. Nail polishes (UV-curable ones are not an exception) should simultaneously demonstrate totally controversial properties: good adhesion with the substrate, high hardness and gloss, as well as scratch and chemical resistance on one side and easy removability by short soaking in some cosmetically acceptable solvent on the other side.

**Figure 15:**
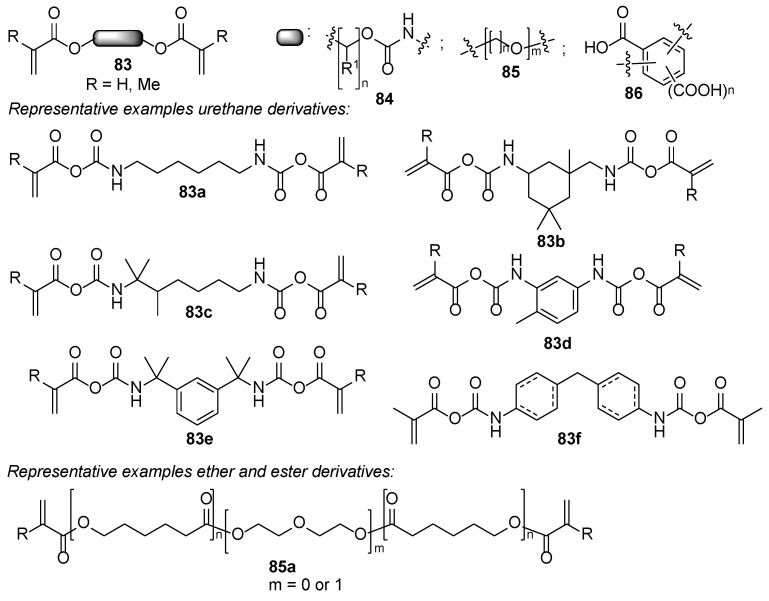
(Meth) acrylate decorated cross-linkable pre-polymers.

**Figure 16:**
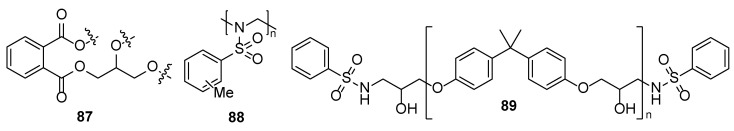
Film-forming polymers.

**Figure 17:**
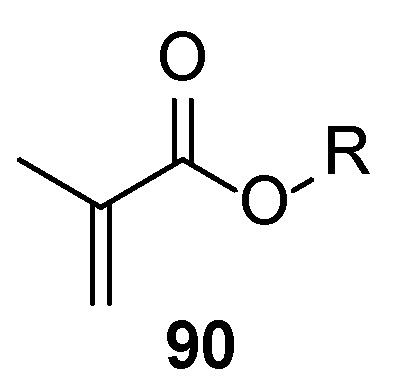
Reactive meth (acrylate) monomers.

A typical UV-curable composition consists of a pre-polymer chain decorated with (meth)acrylate moieties **83**–**86** (Figure 15), a film-forming polymer **87**–**89** (Figure 16), a photoinitiator, a volatile solvent, a (meth)acrylate monomer **90** (Figure 17), and adjuvants for modifying aesthetic and applicability properties [54,55,56]. The pre-polymer bearing at least two (meth)acrylate moieties typically is polyurethane, polyether [54,55], polyester derivatives, or benzene dicarboxylic acid derivatives [57].

Regarding film-forming polymer, it should not be decorated with reactive double bonds. These polymers may be of both natural and synthetic origin, including various modified carbohydrates, polyurethanes, polyacrylates, alkyd resin, resins from aldehyde condensation products, plant-nature resins like dammars, elemi, benzoins, etc. [54,55]. Contrary to synthetic polymers, natural resin may be a mixture of various low molecular compounds, e.g., triterpenoids, which form a sticky substance.

**Figure 18:**
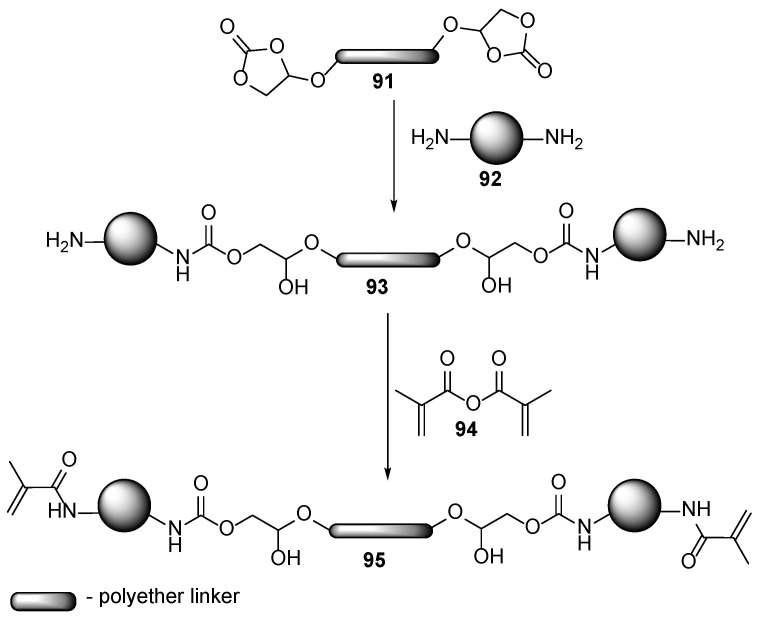
Synthesis of isocyanate-free polyurethane oligomers.

Although urethane-based polymers have widely demonstrated their unlimited applications, recently their utilization has been under discussion due to plausible hazardous health effects caused by the presence of unreacted cyanate residues in the final formulations. This issue is even more serious when the products are used in nail products, as some small-molecule residues from the formulations and even the cured film may penetrate the human body, and some volatile compounds may be inhaled during the application. Thus, the importance of the non-isocyanate acrylic functional polyurethane oligomers [29] synthesized via an amine-cyclic carbonate **94** route (Figure 18) [58] increases.

**Figure 19:**
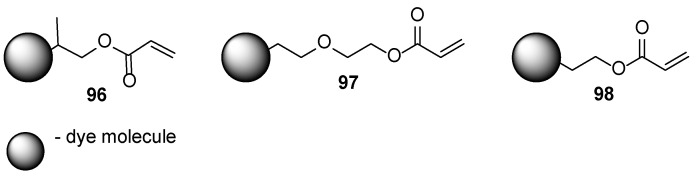
Dye-modified acrylate monomers for preparation coating with covalently bonded dyes.

Various esters of the (meth)acrylic acid are used as reactive monomers [55,56]. Recently it has been provided to functionalize the dyes with acrylate moieties leading to the acrylates **96**–**98**; typically, the dyes are added as small molecular compounds. The main disadvantage of such dyes is plausible migration from the film during the wearing (Figure 19) [59,60]. It is expected that during the cross-linking, these dye-derived monomers are fixed in the polymer film. The curable linker is usually introduced through hydrolytically stable ethereal or sulfoxide moiety. Such an approach is used not only for classical dyes but also for elaborate fluorescent nail polishes [61].

Although there is a lack of powerful methods for studying new nail polishes due to the unique composition of the nail, which cannot be fully reduplicated by a man-made artificial material, there are several general trends from polymer science that may be used for anticipating and extrapolating the properties of the nail polishes. Firstly, a strong correlation between the removability of the nail polish and the gel fraction is observed: the soaking time required for the removal of the layer reduces with a smaller amount of the gel fraction. It is observed that a nitrocellulose or cellulose acetate butyrate additive to the curable acrylate compositions remarkably reduced the soaking time [62]. It is expected that the cellulose derivatives act as keels, thus hindering the formation of a uniform three-dimensional polyacrylate network.

Secondly, the scratch resistance could be characterized by surface abrasion resistance. Cellulose acetate butyrate and sucrose benzoate additives (up to 3%) may improve these properties even twice. The same result may be achieved with a 1% silica additive. It is speculated that these compounds could fill the gaps between the polymer chains, thus acting as surface additives [62].

**Figure 20:**
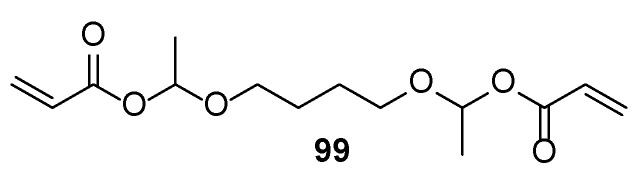
Structure of an acetal moiety containing acrylate.

To improve the curability of the compositions where UV curing is not effective enough, dual UV- and moisture-curable compositions are available: siloxane and isocyanate groups can undergo water-mediated polymerization [63]. Similarly, some compositions use acetal group-decorated acrylates **99** (Figure 20), which, in the presence of cationic photoinitiator PDST, undergo decomposition leading to acrylic acid derivatives found in traditional compositions. Thus, such formulations could be considered as a safer alternative for them [64].

**Figure 21:**
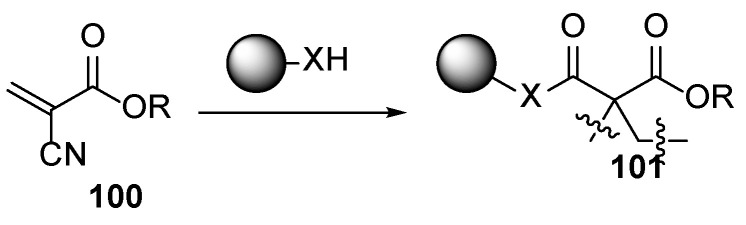
Cyanoacrylates as bifunctional binders.

The improved acrylate compositions also contain cyanoacrylates **100** (Figure 21) which act as a bifunctional binder—firstly, the acrylate moiety is involved in a classical UV polymerization process, and secondly, the cyano moiety may undergo an anionic polymerization with alkyl amines. The main advantage of such films **101** could be the deepening of the polymerization also after interrupting the UV irradiation [65,66].

Additionally, regarding the wearability of the products, the development is going in two opposite directions. On the one hand, the compositions with improved adhesion, thus leading to longer wearing time, are elaborated. On the other hand, to facilitate removing the films from the nail surface and avoid soaking the nails and fingers in organic solvents, compositions with enhanced pealability are introduced.

Besides acrylate or epoxide alone UV-curable compositions, formulations involving both acrylate and epoxide moieties are elaborated, too. Some of such compositions have been postulated as “nail friendly” due to moderate adhesion to the surface. Such compositions use photoinitiators as a mixture of several compounds [53].

**Figure 22:**
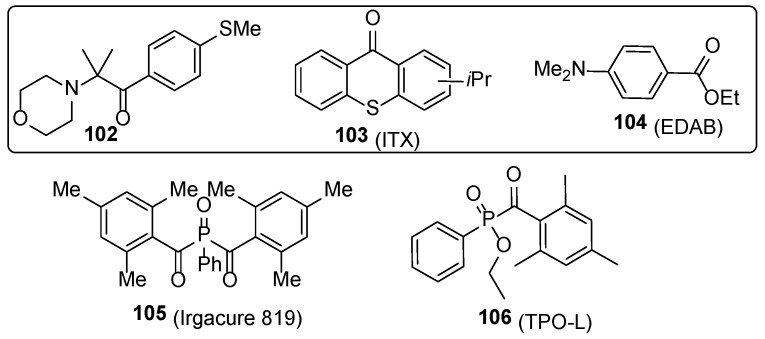
Synergistic compositions of the photoinitiators.

**Figure 23:**
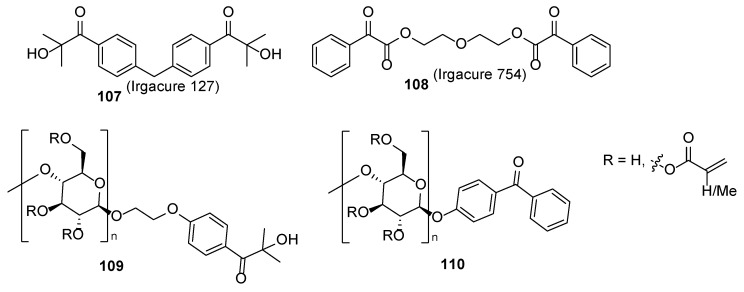
Photoinitiators with increased molecular weight.

One could imagine that the main ingredients for a good nail polish are the film-forming agents. On the other hand, a light-mediated free radical polymerization process occurs on the surface of the nails. To bypass the plausible drawbacks during the UV curing (see part “Acrylate chemistry”), compositions of various photoinitiators are explored. Such compositions may consist of ketone **102**, ITX **103**, and EDAB **104** [67] or TPO-L **105**, ITX **103**, and commercial tertiary amine Ebecryl P115 [30] (Figure 22). The composition consisting of Irgacure 819 (**105**), ITX (**103**), and EDAB (**104**) is established for ultra-fast drying nail polishes—within 5–6 s the layer meets hardness 3H [67]. High molecular photoinitiators **107** and **108** (Figure 23) are developed for reducing (up to 200-fold in comparison with other initiators) the release of photoinitiator-derived low molecular volatile by-product from the nail polish films [68]. An alternative to low molecular photoinitiators is covalent bonding of the initiator to the macromolecular systems bearing polymerizable acrylate units (structures **109** and **110**); thus, the amount of plausible unreacted free photoinitiator in the system is reduced [57]. This film-forming system is used for elaborating environmentally friendly systems where volatile organic solvents are replaced with nano-silica sol. These compositions are characterized by excellent coating properties, abrasion resistance, and drying properties [69].

**Figure 24:**
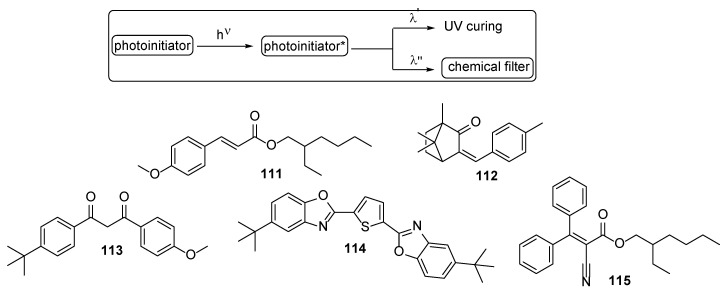
General representation for chemical filters as reducers of the heat exotherms derived during UV curing. Structures of some studied chemical filters.

It is well known that during the polymerization process an exothermic reaction occurs. When the formulations contain various additives and glitters, it might be that the dissipation of the heat is not quick enough and discomfort for the client may occur. Thus, chemical filters that may reduce the exotherms are studied. The role of these compounds may be explained as follows: it is expected that the photoinitiators will dissociate in more than one wavelength region, but the chemical filters **111**–**115** will absorb some range of the actinic radiation, but another region will not be absorbed and is used for mediating UV curing (Figure 24) [63].

With the reduction in the usage of volatile organic compounds, solvent-free compositions of acrylate-epoxide-based formulations are developed. Typically, such dispersions in water are stabilized by non-ionic surfactants (like fatty acid polyethylene glycol derivatives) or anionic surfactants (like ether sulphate-type compounds). Besides, the composition may contain thiol derivatives for increasing adhesion [70]. Within the last observation, the polymers obtained through thiol-ene chemistry turn on particular interest, as these structures contain thio-moieties in remarkable amounts; thus, the adhesion could be improved in comparison to classical acrylate systems.

### 3.2. Bio-Based Acrylates

The bio-based compositions introduce green deal and green chemistry principles also in the cosmeceutical industry as well as increase bio-components in such products as nail polishes, which classically do not contain huge amounts of biodegradable, nature-derived components.

**Figure 25:**
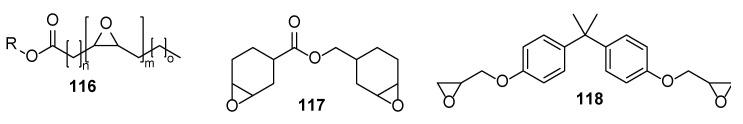
Epoxide derivatives for application in fatty acid-based nail polishes.

**Figure 26:**
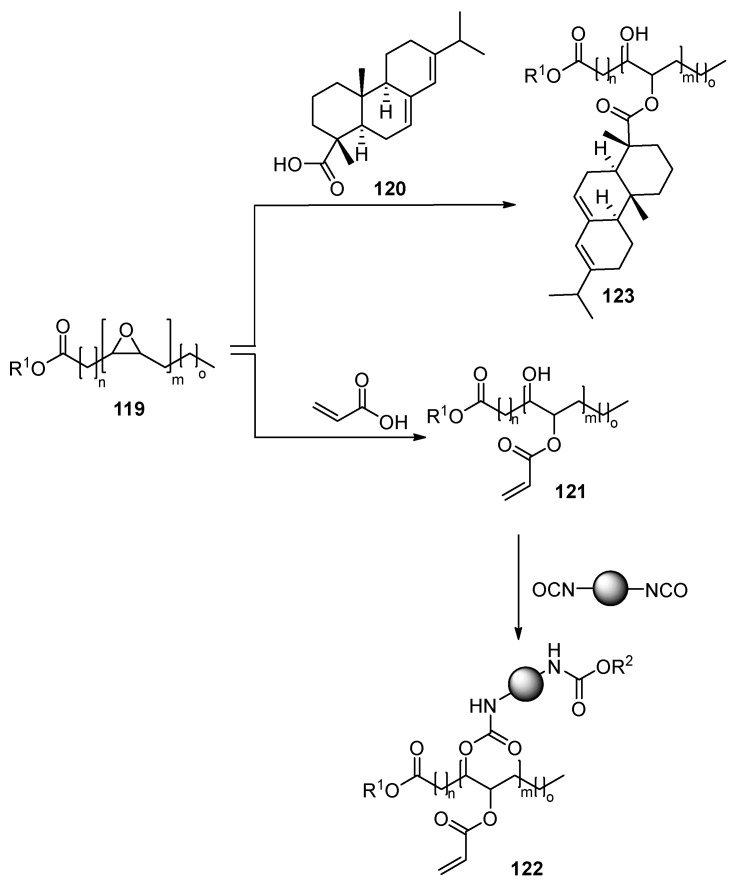
Fatty acid-derived UV-curable nail coatings.

One of the most desirable sources for the bio-based nail polishes would be the introduction of the modified fatty acid sources in the composition of the nail polish. Typically, such compositions contain natural or semi-synthetic epoxy fatty acids with general structure **116**, where R is triglyceride residue, an alkyl or allyl group, and a small molecular epoxide, e.g., compounds **117** or bisphenol A (**118**). The main advantage of epoxide-based layers is the formation of dry film, contrary to traditional acrylate-based films, which, due to incomplete polymerization, tend to form sticky residue on the surface (Figure 25) [71]. However, this problem may be solved via urethane compositions: a vegetable oil-derived acrylate moiety containing pre-polymers is synthesized through treatment of epoxidized soybean oil **119** with acrylic acid [72] or rosin **120** [29]. The acrylate derivative **121** is further subjected to the synthesis of urethanes **122** (Figure 26) [73,74]. Later the synthesized pre-polymer is UV-cross-linked, leading to a film with hardness 4H and toughness over 300% [73]. Some of the elaborated vegetable oil-based compositions contain more than 40% of bio-based renewable raw materials. Even more, such formulations are investigated as emulsions in water; thus, the usage of volatile organic solvents is reduced [72]. The hardness of such coating does not differ whether a UV-mercury or UV-LED source was used for hardening the layer [74]. Another urethane-acrylate-based nail polish composition involves the usage of drying vegetable oils (4–6%). The double bonds of the vegetable oils via radical-induced oxidation processes are grafted into the main polymer chain [75].

**Figure 27:**
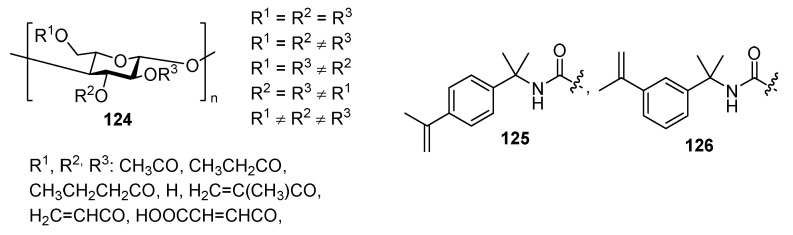
Cellulose-derived films for UV-curable nail polishes.

Bio-based acrylate-type UV-curing nail polish can be derived from cellulose **124** (Figure 27). A typical composition may contain up to 95% of modified cellulose ester along with low-molecular cross-linking agent **125** or **126** containing ethylene, diene, styrene, vinyl ether, or acrylic acid moiety. Such cellulose-based coatings are characterized by good solvent resistance, and the pencil hardness for the surface is equal to or greater than H [76].

**Figure 28:**
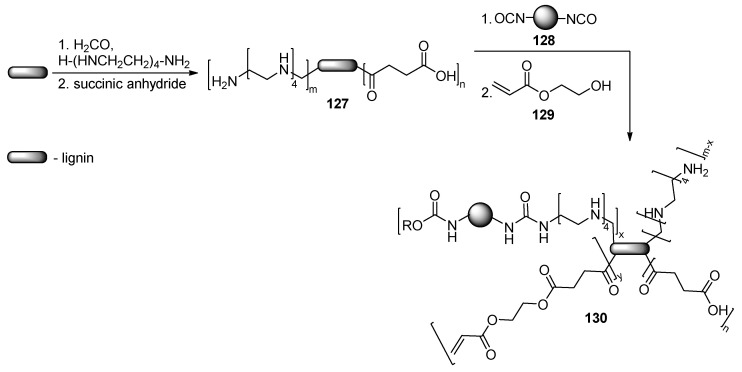
Lignin-based films for nail polishes.

Biomass-based UV-curable films may be prepared from polyamino and polycarboxy group modified lignin **127** (Figure 28). The treatment of such modified lignin with diisocyanate **128** and hydroxyethyl acrylate **129** leads to the pre-polymer **130**, which may be later UV cured [77].

**Figure 29:**
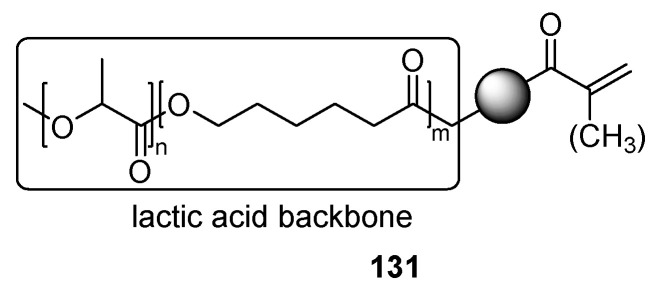
Polylactic acid derived UV-curing coatings.

A bio-based polymer backbone for nail coatings is obtained by modification of poly-lactic acid-ε-caprolactone **131** with acryloyl groups (Figure 29). Later, such a backbone may be cross-linked with multifunctional acrylates, leading to coatings with improved adhesion [78].

The bio-based motif may appear not only as a carrier for the acrylic moieties but also as an ingredient for preparing the polyurethane that is introduced in the formulation of nail polishes. Such an example involves, e.g., polyurethanes obtained from isophorone diisocyanate, hydroxyethyl acrylate, and cellulose-derived polyethylene succinate [79].

The biomass-derived acrylates, contrary to petroleum-based acrylates, are characterized by an increased number of free hydroxyl groups in the structure. Zarenanshakari and Mannari postulate that the increased hydroxyl number could facilitate a hydrogen bonding network with the substrate [29]. Thus, it can be expected that these compounds could be valuable both from the green chemistry perspective and as ingredients for nail polish compositions with improved adhesion. The rosin derivative turns into a particular interest as rosin itself is a well-known ingredient for various adhesives [80].

## 4. Additives for Improved Adhesion

To enhance the adhering between the nail surface and the coating and to ensure good wearability of the nail coatings, various tools are applied. All the applied methods may be allocated in the following groups: (1) various techniques for pretreating the nail surface prior to the coating being applied and (2) improving the adhesive properties of the nail polish [81].

The pretreatment techniques involve both mechanical processing and chemical treatment of the nail surface. Chemical treatment may involve the application of various primers before the base layer. Regarding the primers, three types are known: (1) acid-based primers type I (methacrylic acid), (2) acid-based primers type II (all other acids except methacrylic acid), and (3) non-acid primers (the compounds do not contain acids). The main task of these compounds is to dehydrate and to wash the lipid particles from the nail surface [81]. A different solution is provided by Mididoddi et al. [82]: tartaric acid is reported as a compound for improving bioadhesion. Herein it is believed that instead of forming intermolecular bonds, tartaric acid acts as a surface modifier, and the dorsal layer is disrupted as a result. The roughness of the nail surface and the availability of the surface for intermolecular hydrogen bonding are increased. This technique is used for ketoconzazole-containing hydroxypropyl cellulose films. The application of tartaric acid’s exfoliating properties [83] could also prove useful for UV-curing compositions, either as one of the ingredients or as a pre-treatment before applying the nail polish layer.

The compounds designed for improving adhesion involve adhesion promoters, pre-bond compounds, and coupling agents. Typically, these compounds contain a photoreactive group and an additional functional group that may interact with the keratinous surface through ion-type or hydrogen bonds.

**Figure 30:**
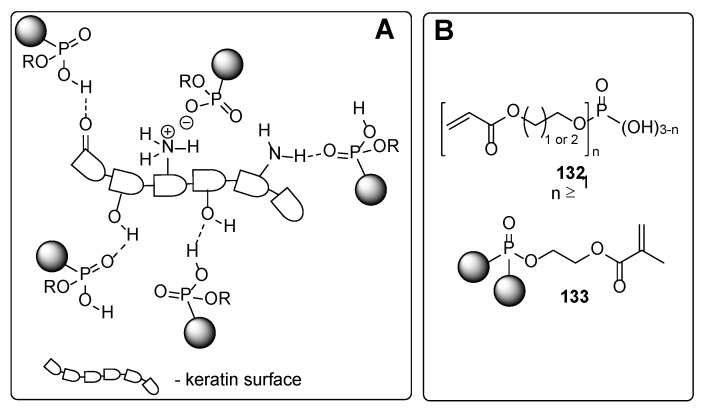
Phosphate-derived adhesives: plausible interaction with the nail surface (**A**) and the adhesives (**B**).

**Figure 31:**
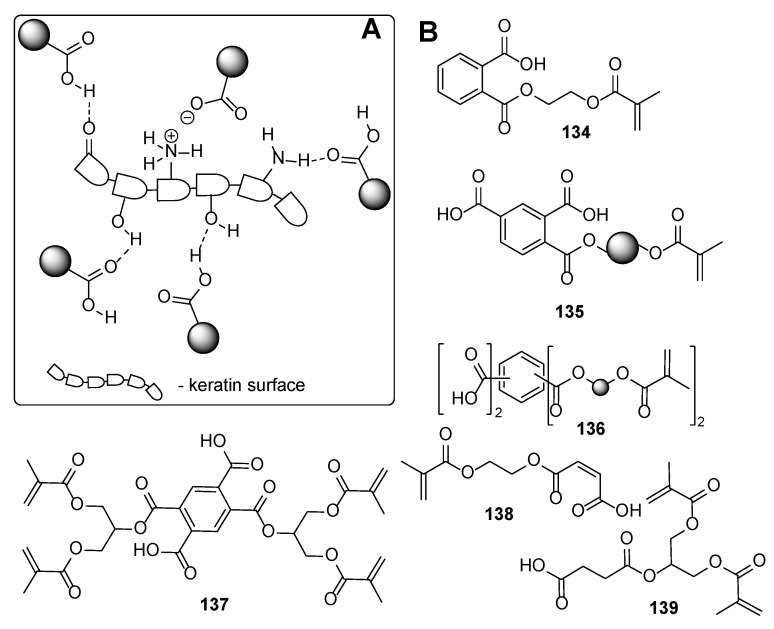
Acid group-containing compounds: plausible interaction with the nail surface (**A**) and the adhesives (**B**).

**Figure 32:**
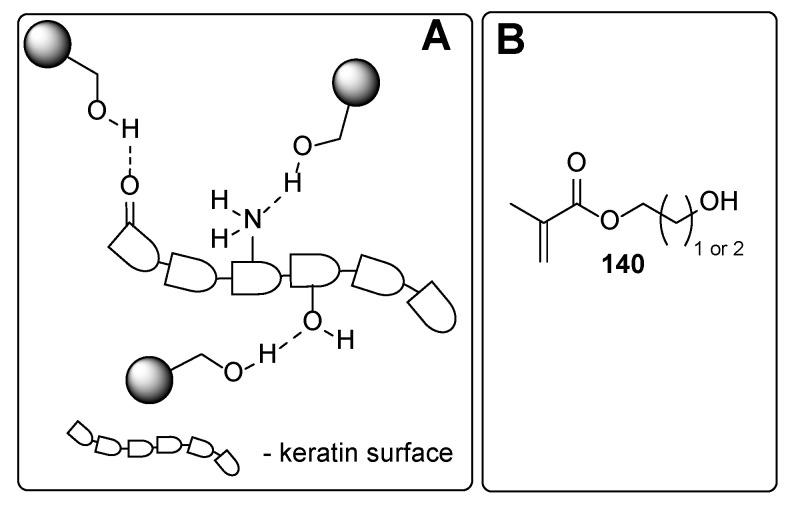
Alcohol moiety-containing derivatives: plausible interaction with the nail surface (**A**) and the adhesive (**B**).

**Figure 33:**
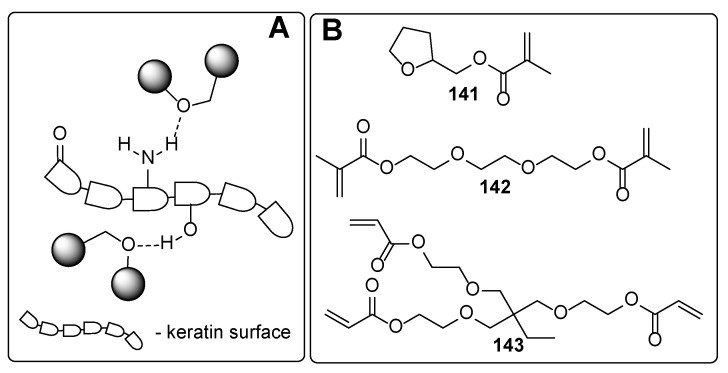
Etheral moiety-containing derivatives: plausible interaction with the nail surface (**A**) and the adhesives (**B**).

**Figure 34:**
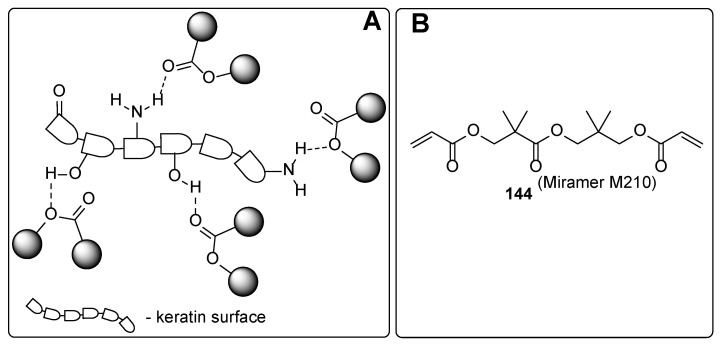
Ester derivatives: plausible interaction with the nail surface (**A**) and the adhesive (**B**).

**Figure 35:**
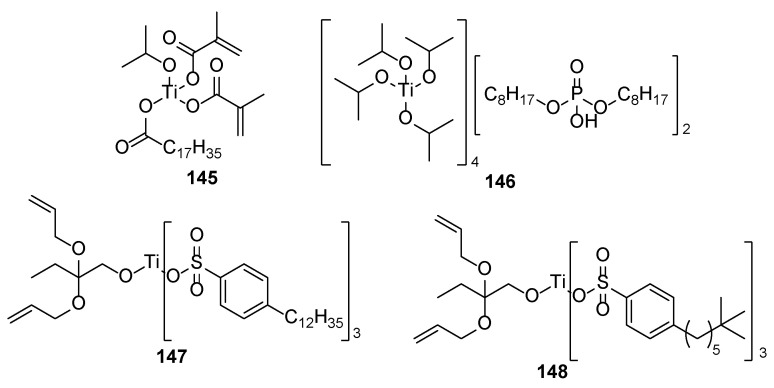
Organotitanium compounds as adhesives.

**Figure 36:**
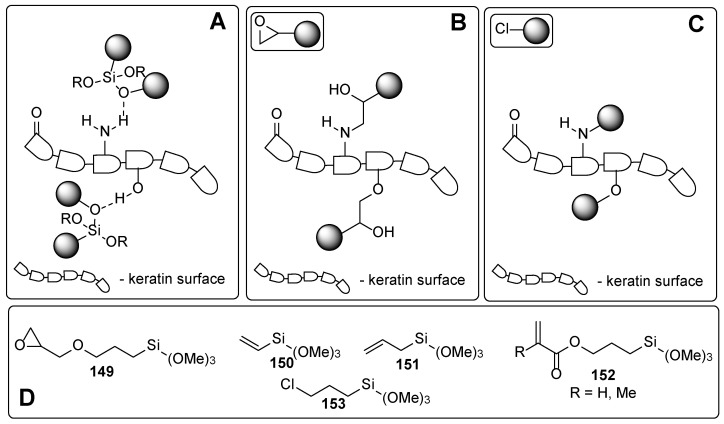
Plausible interactions of the nail surface with various agents: (**A**) hydrogen bond network in silane derivatives; covalent bonding with the epoxide (**B**) and halogenide (**C**) containing adhesives; (**D**) representative structures of various agents.

From the chemistry point, these compounds involve phosphates, carboxylic acids and their derivatives, and ethers. In the following, some representative examples for these compounds are summarized. Among phosphate derivatives (Figure 30), β-hydroxyethyl and β-hydroxypropyl(meth)acrylates **132** and **133** are provided [84,85,86]. Examples of acids (Figure 31) are commercial product Sarbox 500E (**134**) [84,87,88] or phthalic acid half ester **135** [87]. The last one is an aromatic acid methacrylate half ester provided in ethoxylated trimethylolpropane triacrylate—carboxylic acid and anhydride-containing methacrylate oligomer [89]. Other free carboxylic acids **136**–**139** are phthalic [90], pyromellitic [57,90,91], malic [86,90] and succinic acid [90]. The adhesion-increasing units may also bear alcohol **140** (Figure 32) [90,91,92], cyclic **141** [90], and acyclic **142** and **143** [91,93] ethereal (Figure 33) and ester **144** (Figure 34) [86] moieties, as well as phosphate derivatives. Another group of potential adhesives are organotitanium compounds **145**–**148** (Figure 35) [88]. Silane derivatives **149**–**153** (Figure 36) may be useful coupling agents, too. These compounds may contain epoxy, vinyl, and (met)acryl moieties. Besides monomers, various polyorganosiloxane (silicone acrylate polyether) compounds are mentioned [91,93,94,95]. Among those previously mentioned, the base coat may contain aminosilanes and hydroxysilanes, too [95].

A comparative study of various adhesion promoters, 5% additive of (*γ*-methacryloxypropyltrimethoxysilane, dimethacrylate—succinate adduct, dimethacrylate—maleate adduct, phosphate derivatives, *bis*(methacryloyloxyethyl)acrylate) have turned: (1) *γ*-methacryloxypropyltrimethoxysilane increases the maximum polymerization temperature (>90 °C) thus discomfort for the client can be caused; (2) the maleate adduct demonstrated remarkably reduced elongation and improved tensile strength for the film in comparison to other adhesive additives; and (3) *bis*(methacryloyloxyethyl)acrylate showed the highest adhesion strength to aluminum sheets [96].

Most of the previously summarized adhesives can interact with the surface of the nail mainly through hydrogen bonds, like “adhesive-O-H···O=keratin residue”, “adhesive=O···H-O-keratin residue”, and/or “adhesive=O···H-N-keratin residue”. The adhesion properties of the various compounds could organize the following order: phosphates ≈ carboxylic acids > esters ≥ alcohols > ethers. The adhesion properties strongly correlate with the hydrogen bond-forming properties of the adhesive structure. It is clearly demonstrated that phosphates and carboxylic acid derivatives may act both as hydrogen bond donors and hydrogen bond acceptors, leading to a tight intermolecular bond network between the coating and the nail surface. On the contrary, careful analysis of the provided adhesives containing ester, alcohol, or ethereal moiety demonstrated reduced ability for forming the intermolecular network. The esters may interact via two types of hydrogen bonds (“adhesive=O···H-N/H-O-keratin residue” or “adhesive-(R)-O···H-N/H-O-keratin residue”), while the compounds containing ethereal moiety may interact only via “adhesive-(R)-O···H-N/H-O-keratin residue” hydrogen bonds. The alcohol derivatives cannot be classified as superior intermolecular network-forming agents. However, contrary to esters and ethers, they may be involved both as hydrogen bond donors and acceptors.

**Figure 37:**
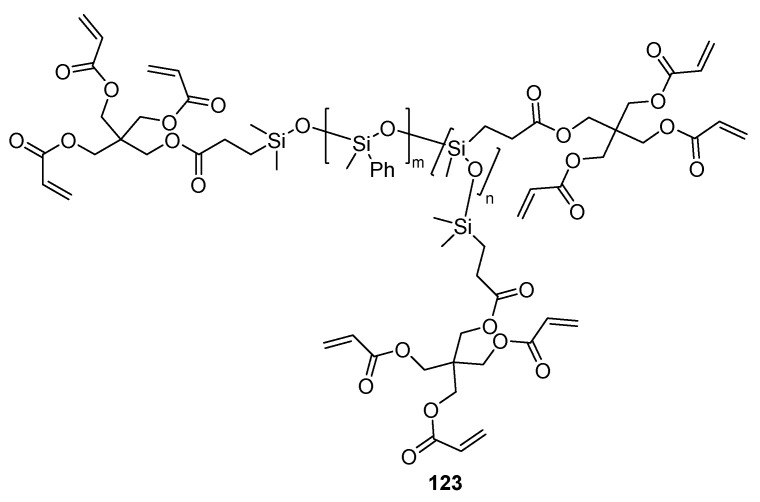
Silicone-based acrylate for promoting pealability of the nail coatings.

The nails are constituents primarily of horny skin cells—proteins named keratin. The secondary structures of the keratin form *α*-sheets, random coils, and *α*-helical regions [97]. The chemical composition analysis of nail keratin has revealed glutamic acid as the major one, followed by cystine, arginine, leucine, and serine [98]. It should be admitted that a part of the amino acids forming the keratin are so-called basic amino acids (like arginine, histidine, and lysine), which ensures “free” amino functionalities on the surface of the protein backbone. Thus, the improved adhesive properties of the phosphates and carboxylic acids among the other studied adhesives may be attributed to their ability to form ionic bonds through the ammonium salts. Although Figure 31, Figure 32, Figure 33, Figure 34, Figure 35, Figure 36 and Figure 37 present only interactions between the amino groups or carboxylic groups (both the carbonyl group and its hydroxyl group) and the adhesive, an additional interaction between the adhesive and the keratin may arise from the hydroxyl groups (in serine) or thiol functionality (in cysteine and methionine).

However, it is expected that in some compositions, additional covalent bonds between the nail surface and the adhesive may form through cleavage of the oxirane ring or alkylation with alkyl chloride.

Besides small-molecular additives, formulations using additional sticky adhesive resin [87], B302 [99], and latex film are known [100]. On the other hand, latex film is involved in the system, which may be removed by simple treatment with warm water [100].

Not only the effectiveness of the adhesion properties should be considered as essential for a perfect nail polish. Due to the liveliness of the nail caused by human habits and lifestyle, when the nail may be subjected to various movements, the nail polish film should be able to obey the transformations. Thus, herein the elasticity and toughness of the layer become crucial. It is provided to analyze these films with the modulus of elasticity as well as elongation and tensile strength. The modulus of elasticity increases >20% when a nitrocellulose additive is used in comparison to acrylate composition without this additive. Similarly, nitrocellulose additive has a positive impact on the elongation, while sucrose benzoate improves the tensile strength [101].

Although one could expect that the client prefer a nail polish with prolonged adhesion, the main inconvenience for the acrylate-based UV-curing nail polishes arises from removing such coatings due to their insolubility in the common organic and even more, cosmeutically acceptable solvents, and mechanical removal may be required. Thus, the innovations are driven in two opposite directions: firstly, to improve the adhesion and secondly, to facilitate the removability. To solve the inconvenience during when pealing off the film is required, the compositions of the nail polishes may be modified with different pro-formed polymers. Some examples involve introducing adipic acid—neopentyl glycol—trimellitic anhydride copolymers, tosylamide—epoxy resin, or styrene—acrylate copolymer in the UV-curable composition of acrylate. It is expected that the pre-polymer inclusions will serve as hurdles for forming polymer networks, but the advantages of the UV-curable nail films are maintained. The authors suggest that these coatings may be removed by simply soaking in acetone, methyl ethyl ketone, or ethanol for less than 5 min [102]. Some other solutions are the introduction of a modified silicon-based acrylate **154** (Figure 37) in the compositions of the nail polishes. It is demonstrated that in these structures, due to the rather lipophilic silicone structure, the surface energy is reduced, thus leading to reduced interaction between the nail surface and the film [103,104,105,106,107,108,109,110].

## 5. Photoinitiator-Free “On Nail” Curing Compositions

**Figure 38:**
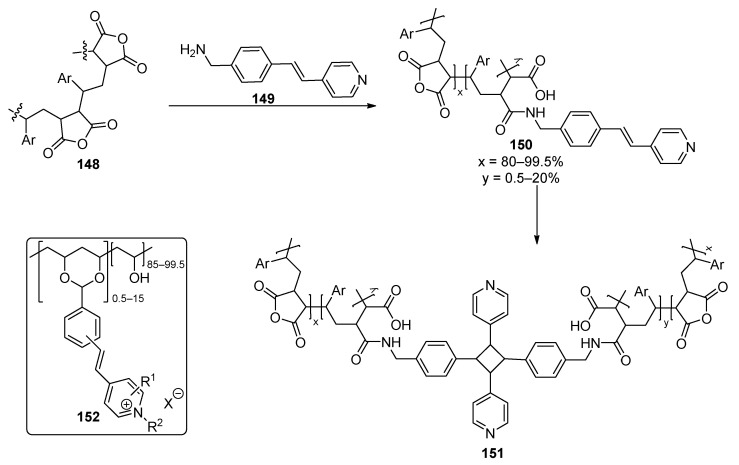
Styrylpiridinium-based derivatives for nail coatings.

A UV-curing composition without additional photoinitiators is based on styrylpyridine derivatives **157** (Figure 38). A classical styrene-maleic anhydride polymer **155** is decorated with a styrylpyridine moiety through an amide bond. Later the obtained material can undergo UV/visible light-mediated cross-linking through a [2+2] cycloaddition reaction. This polymer has demonstrated improved shine, gloss adhesion, and wearability for nail films. To reduce the brittleness of some of these polymer combinations with other traditional polymers like epoxy and acrylic resins, polyurethanes, or cellulose derivatives are elaborated [111]. A similar principle is used also in polyvinyl alcohol polymers **159**, which are decorated with styrylyridine residues. Such compositions improved with additional complexation agents like sodium borate and demonstrate excellent shine and adhesion also during wearing [112].

**Figure 39:**
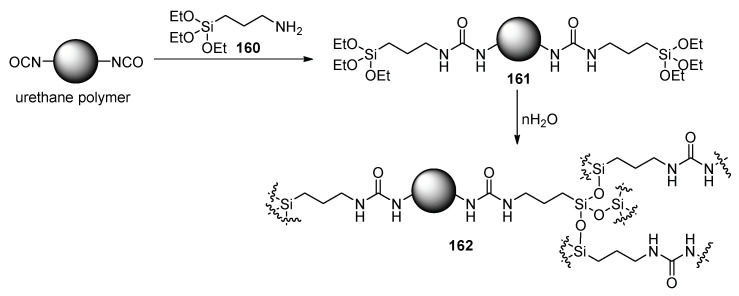
Silicon network-based films.

A promising future for nail enamels is indicated by Peng et al. This perspective foresees modification of urethanes with organosilicium compound **160** and later hydrolysis of the product **161** and condensation leading to silicon network **162**, which forms the film (Figure 39). Such film is characterized by 2H pencil hardness [113]. Although UV-free curing would have a lot of benefits, the current composition requires more than 2 h of drying, which, in the present performance, could be a good alternative for artificial nails, but the polymerization directly on the nail plate does not seem reasonable.

## 6. Other Nail Products

Besides classical nail polishes, nail stickers for the beauty industry are developed, too. Such products are prepared by applying several UV-curing layers one by one, leading to a semi-cured state. When the stickers are applied on the nails, UV or visible light mediates the hardening process [114]. Although the first associations for nail polishes are mainly related to aesthetic purposes and demonstration of individuality, some studies have turned UV-curable nail polishes into an attractive drug delivery system for topical treatment of various nail diseases. Contrary to traditional nail polish compositions used in cosmetics, the formulations used as drug delivery systems are characterized by remarkably simpler systems consisting only of two (ethyl methacrylate and diurethane dimethacrylate) [115] or three (ethyl methacrylate, isobornyl methacrylate, or hydroxyethyl methacrylate) [116] monomers. The corresponding methacrylates are preferred over the acrylates due to their reduced toxicity and sensitizing [116]. These compositions were applied for encapsulation of amorolfine hydrochloride or terbinafine hydrochloride [115,116]—both known as medicines for treating fungal nail diseases. The maximum release of the medicine from the cured film as well as permeation in the nail is reached within 15 days. The restricting criteria for such products are (1) the limited solubility of the pharmaceuticals in the monomers, thus a co-solvent like ethanol may be required, and (2) oxygen-inhibited curing of the monomers leading to the sticky surface that is later wiped with some appropriate solvent. The insufficient polymerization led to incomplete encapsulation of the active ingredients and reduced concentration of the drug in the film. However, the incomplete polymerization caused a reduction in the active component in the film that could be solved with an additional UV-curable (even decorative) film.

From the application in cosmetics view, the odor of nail care products caused by the organic solvents and methacrylic acid may raise unpleasant senses. Thus, the products with improved scent would be valuable. Bednarczyk et al. have developed a composition improved with a citrus fragrance—R-(+)-limonene. Similarly, the previously described pharmaceutical delivery systems for nail diseases, wherein the limonene is added to the mixture of the acrylates and later, during the UV curing, are encapsulated among the polymer chains. The limonene is slowly released from the film for up to a week. Even more, the limonene additive can reduce the curing temperature by 20 degrees [27], which is essential for reducing plausible unpleasant heat-caused feelings during the curing process. Moreover, such limonene-enriched films have demonstrated antibacterial activity against *E. coli* and fungicidal properties against *Candida albicans* [27]. Thus, essential oils in improved nail polishes, besides their classical aesthetic functionalities, could serve as a platform for slight protection against bacteria and fungi. Additionally, this could be a tool for improving sensory properties (scent) for those polymers obtained by thiol-ene chemistry for suppressing the aroma of thiols.

## 7. Conclusions

The development of nail polishes had a long way to go, starting from fragile, nature-derived coatings to acrylate UV-curing compositions with good wearability. The modern UV-curing nail polish is a complicated composition consisting of film-forming polymers, pre-polymers, acrylate monomers, and additives. The additives are responsible for good adhesion with the surface of the nail, forming a simultaneously hard and flexible film, as well as aesthetic effects like colors, shine, gloss, etc. The general compositions are well established. Thus, all the efforts nowadays are devoted to polishing the composition to perfection. These issues include studies of promoters with improved adhesion between the nail surface and the polymer film from one side and compositions with improved pealability when the product should be removed from another side. The composition of photoinitiators is crucial to ensure full polymerization without forming a sticky layer of low-molecular polymerization products: on one hand, the increase in the concentration would be a solution; on the other, the polymerization typically is an exothermic process, and the increase in the temperature should be carefully monitored. During the wearing of the nail polish, it is expected that some low molecular compounds like dyes or photoinitiators could release from the film; thus, polymer-bonded alternatives are of interest nowadays.

Another course of interest is complete or partial replacement of classical petroleum-based acrylates with nature-derived compounds. Currently, main efforts are devoted to fatty acid derivatives; however, depending on the unsaturation of the fatty acids, the properties of the final product may change in a rather wide range. Acrylate derivatives are studied also for carbohydrates and lignin. The up-to-date tendencies will increase the ratio of natural components in the nail polish; thus, the health issues caused by low molecular acrylates could be reduced, as well as the microplastics that arise during wearing. Acrylate-modified highly unsaturated oils in combination with natural adhesives could lead to human- and nature-friendly nail polishes with acceptable mechanical and aesthetic properties.

## Figures and Tables

**Table 1:** Overview of various UV-curable systems.

Component	Advantages	Disadvantages
Acrylate-based compositions		
(Meth)acrylate derived monomers and pre-polymers	Available both petroleum-based and nature-derived components Widely available	Side reaction during polymerization may lead to large distribution of polymer chain length and the properties of the film Incomplete polymerization may lead to sticky layers which should be removed with suitable solvents Occupational disease and allergy risk
Photoinitiators	Wide diversity of structures Exposure to UV light, thus the systems are compatible with various colors	Type I photoinitiators are sensitive towards oxygen. As a result, incomplete polymerization may occur
Film-forming agents	Various natural and synthetic polymers Wide diversity	When polyurethanes are used, the risk of the presence of unreacted isocyanate should be taken into consideration
Epoxide-based compositions		
Epoxide moiety bearing monomers and pre-polymers	Available both petroleum-based and nature-derived compounds	Epoxides due to their high reactivity can undergo various side reactions with other nucleophiles
Photoinitiators	Available both Lewis and Bronsted photoacids	Strong acids may be elaborated during the polymerization process
Thiole—ene compositions		
Acrylates	The same as in mentioned at acrylate-based compositions	
Thiols	Available both natural and synthetic The impact of oxygen on the polymerization process is reduced	Unpleasant odor especially for the low molecular compounds
Photoinitiators	As the thiols may act as antioxidants the impact of oxygen on the effectiveness of the type I photointitators is reduced	

## Abbreviations

The following abbreviations are used in this manuscript:

EDAB	Ethyl 4-(dimethylamino)benzoate
DNA	Deoxyribonucleic acid
ITX	Isopropylthioxanthone
LED	Light-emitting diode
PDST	(4-Pheylthiophenyl)diphenylsulphonium triflate
UV	Ultraviolet
TPO-L	Ethyl phenyl(2,4,6-trimethylbenzoyl)phosphinate
